# Rapid methods for the detection of foodborne bacterial pathogens: principles, applications, advantages and limitations

**DOI:** 10.3389/fmicb.2014.00770

**Published:** 2015-01-12

**Authors:** Jodi Woan-Fei Law, Nurul-Syakima Ab Mutalib, Kok-Gan Chan, Learn-Han Lee

**Affiliations:** ^1^Jeffrey Cheah School of Medicine and Health Sciences, Monash University MalaysiaSelangor Darul Ehsan, Malaysia; ^2^School of Science, Monash University MalaysiaSelangor Darul Ehsan, Malaysia; ^3^UKM Medical Molecular Biology Institute (UMBI), UKM Medical Centre, Bandar Tun RazakKuala Lumpur, Malaysia; ^4^Division of Genetics and Molecular Biology, Institute of Biological Sciences, Faculty of Science, University of MalayaKuala Lumpur, Malaysia

**Keywords:** foodborne, pathogens, rapid, detection, PCR, NASBA, LAMP

## Abstract

The incidence of foodborne diseases has increased over the years and resulted in major public health problem globally. Foodborne pathogens can be found in various foods and it is important to detect foodborne pathogens to provide safe food supply and to prevent foodborne diseases. The conventional methods used to detect foodborne pathogen are time consuming and laborious. Hence, a variety of methods have been developed for rapid detection of foodborne pathogens as it is required in many food analyses. Rapid detection methods can be categorized into nucleic acid-based, biosensor-based and immunological-based methods. This review emphasizes on the principles and application of recent rapid methods for the detection of foodborne bacterial pathogens. Detection methods included are simple polymerase chain reaction (PCR), multiplex PCR, real-time PCR, nucleic acid sequence-based amplification (NASBA), loop-mediated isothermal amplification (LAMP) and oligonucleotide DNA microarray which classified as nucleic acid-based methods; optical, electrochemical and mass-based biosensors which classified as biosensor-based methods; enzyme-linked immunosorbent assay (ELISA) and lateral flow immunoassay which classified as immunological-based methods. In general, rapid detection methods are generally time-efficient, sensitive, specific and labor-saving. The developments of rapid detection methods are vital in prevention and treatment of foodborne diseases.

## Introduction

Foodborne diseases have become a major public health problem worldwide due to the significantly increased incidence of foodborne diseases over the last 20 years (Oliver et al., 2005). Although it is difficult to estimate the global incidence of foodborne diseases as some of the cases are under-reported especially in developing countries, but the increased incidence of foodborne diseases were reported in many parts of the world (Van de Venter, 2000). For instance, the outbreak of foodborne disease in Taiwan increased rapidly from 121 in 1995 to 177 in 1996 and since then the incidence keep rising (Chiou et al., 2000). According to report from Centers for Diseases Control and Prevention (CDC), approximately 48 million people in the United States get ill, 128000 people are hospitalized and 3000 people die annually due to foodborne diseases despite United States has the safest food supplies in the world (Oliver et al., 2005; Centers for Disease Control and Prevention, 2011). In addition, about a quarter of the population is at a higher risk for foodborne diseases nowadays (Oliver et al., 2005).

Generally, foodborne diseases are caused by the consumption of food or water contaminated with pathogens or their toxins. Pathogens that caused foodborne diseases are often referred as foodborne pathogens and they include bacteria, viruses, fungi and parasites (Zhao et al., 2014). There are 31 identified foodborne pathogens in the United State and it is estimated that viruses are the primary causes of illnesses whereas bacteria are the primary causes of hospitalizations and deaths (Scallan et al., 2011). The common foodborne pathogens which are responsible for most of the foodborne disease outbreaks are *Listeria monocytogenes, Escherichia coli* O157:H7, *Staphylococcus aureus, Salmonella enterica, Bacillus cereus, Vibrio* spp., *Campylobacter jejuni, Clostridium perfringens*, and Shiga toxin-producing *Escherichia coli* (STEC) (Oliver et al., 2005; Scallan et al., 2011; Zhao et al., 2014).

The increasing amounts of street foods and the increasing demand for minimally processed ready-to-eat products have begun to concern public health agencies on food safety assurance (Lee et al., 2014). Foodborne pathogens are present in various foods such as fruits, vegetables and ready-to-eat products which are consumed without any further treatment (Chung et al., 2010; Lee et al., 2014). This may lead to foodborne diseases if food safety issues are not taken into consideration. Also, foodborne diseases are often associated with the consumption of raw or undercooked foods such as seafood, meat and poultry (Wingstrand et al., 2006; Rosec et al., 2012; Omurtag et al., 2013). It is essential to analyze the food for the presence of foodborne pathogens in order to ensure a safe food supply and to minimize the occurrence of foodborne diseases.

The conventional methods for detecting the foodborne bacterial pathogens present in food are based on culturing the microorganisms on agar plates followed by standard biochemical identifications (Mandal et al., 2011). Conventional methods are usually inexpensive and simple but these methods can be time consuming as they depend on the ability of the microorganisms to grow in different culture media such as pre-enrichment media, selective enrichment media and selective plating media. Usually conventional methods require 2 to 3 days for preliminary identification and more than a week for confirmation of the species of the pathogens (Zhao et al., 2014). Conventional methods are laborious as they require the preparation of culture media, inoculation of plates and colony counting (Mandal et al., 2011). Furthermore, conventional methods may be limited by their low sensitivity (Lee et al., 2014). False negative results may occur due to viable but non-culturable (VBNC) pathogens. The failure to detect foodborne pathogens would increase the transmission risk of pathogens.

Recently, different rapid methods with high sensitivity and specificity have been developed to overcome the limitations of conventional methods for the detection and identification of foodborne pathogens. Furthermore, researchers are still developing novel methods with improvements in terms of rapidity, sensitivity, specificity and suitability for *in situ* analysis and distinction of the viable cell (Zhao et al., 2014). Rapid detection methods are important, particularly in food industry, as they are able to detect the presence of pathogens in raw and processed foods immediately. Rapid methods are also sensitive enough to detect pathogens that present in low numbers in the food. Sensitivity is important because a single pathogen present in food has the risk to cause infection. Rapid methods are more time-efficient, labor-saving and able to reduce human errors (Mandal et al., 2011). Nevertheless, each of the rapid method has its own advantages and limitations. Generally, rapid detection methods are categorized into nucleic acid-based, biosensor-based and immunological-based methods (Zhao et al., 2014). This review examines these recent rapid detection methods and their applications in foodborne bacterial pathogens detection and along with their advantages and limitations.

## Nucleic acid-based methods

Nucleic acid-based methods operate by detecting specific DNA or RNA sequences in the target pathogen. This is done by hybridizing the target nucleic acid sequence to a synthetic oligonucleotide (probes or primers) which is complementary to the target sequence (Zhao et al., 2014). There are many bacterial pathogens such as *Clostridium botulinum, Vibrio cholerae, Staphylococcus aureus*, and *Escherichia coli* O157 which produced toxins that caused foodborne diseases (Singh et al., 2001; Akbulut et al., 2004; Fusco et al., 2011; Son et al., 2014). The toxin-related genes in these pathogens can be detected by nucleic acid-based methods (Zhao et al., 2014). Furthermore pathogens showing ambiguous phenotypic characteristics such as hippurate negative *Campylobacter jejuni* strains can be identified and confirmed by nucleic acid-based methods (Adzitey et al., 2012). Nucleic-acid based methods detect the specific genes in the target pathogens, therefore preventing ambiguous or wrongly interpreted results. The recent nucleic acid-based methods described are simple polymerase chain reaction (PCR), multiplex polymerase chain reaction (mPCR), real-time/quantitative polymerase chain reaction (qPCR), nucleic acid sequence-based amplification (NASBA), loop-mediated isothermal amplification (LAMP) and microarray technology.

### Polymerase chain reaction (PCR)

One of the most commonly used molecular-based method for the detection of foodborne bacterial pathogens is polymerase chain reaction (PCR). PCR was invented about 30 years ago and it allows the detection of a single bacterial pathogen that present in food by detecting a specific target DNA sequence (Velusamy et al., 2010). PCR operates by amplifying a specific target DNA sequence in a cyclic three steps process (Mandal et al., 2011). Firstly, the target double-stranded DNA is denatured into single-stranded DNA at high temperature. Then, two single-stranded synthetic oligonucleotides or specific primers which are the forward and reverse primer will anneal to the DNA strands. This is followed by the polymerization process whereby the primers complementary to the single-stranded DNA are extended with the presence of deoxyribonucleotides and a thermostable DNA polymerase. The PCR amplification products are visualized on electrophoresis gel as bands by staining with ethidium bromide (Zhao et al., 2014). PCR have been used in the detection of numerous foodborne pathogens like *Listeria monocytogenes*, *Escherichia coli* O157:H7, *Staphylococcus aureus, Campylobacter jejuni, Salmonella* spp. and *Shigella* spp. (Cheah et al., 2008; Lee et al., 2008; Alves et al., 2012; Chiang et al., 2012; Zhou et al., 2013).

### Multiplex PCR (mPCR)

Multiplex PCR offers a more rapid detection as compared to simple PCR through the simultaneous amplification of multiple gene targets. The basic principle of mPCR is similar to conventional PCR. However, several sets of specific primers are used in mPCR assay whereas only one set of specific primers are used in conventional PCR assay. Primer design is very important for the development of mPCR, as the primer sets should have similar annealing temperature in order to produce a successful mPCR assay (Zhao et al., 2014). Besides, the concentration of primers is also important in mPCR. This is because interaction may occur between the multiple primer sets in mPCR that results in primer dimers, thus, the concentration of primers may need to be adjusted to ensure the production of reliable PCR products (Zhao et al., 2014). Other important factors for a successful mPCR assay include the PCR buffer concentrations, the balance between magnesium chloride and deoxynucleotide concentrations, the quantities of DNA template, cycling temperatures and Taq DNA polymerase (Markoulatos et al., 2002; Cheah et al., 2008; Khoo et al., 2009).

Previously, mPCR was used to detect around two to three pathogens only. Now, mPCR is more advanced and it can detect up to five or more pathogens simultaneously. Chen et al. (2012) had carried out mPCR for the simultaneous detection of *Salmonella* Enteritidis*, Staphylococcus aureus, Shigella flexneri, Listeria monocytogenes*, and *Escherichia coli* O157:H7 using five pairs of primers targeting invasion protein (*invA*), 16S rDNA, invasion plasmid antigen H (*ipaH*), listeriolysin O (*hlyA*) and intimin (*eaeA*) gene, respectively. Ryu et al. (2013) had developed a novel mPCR assay which is able to detect and discriminate six *Listeria* species simultaneously in one tube with high accuracy. The *Listeria* species that were successfully identified are *Listeria monocytogenes, Listeria grayi, Listeria ivanovii, Listeria innocua, Listeria welshimeri*, and *Listeria seeligeri*. The mPCR detection limit was 7.58 × 10^4^ copies for mixed genomic DNA.

Further improvements of mPCR include the development of a novel GeXP-PCR by Zhou et al. (2013) for the simultaneous detection of six foodborne bacterial pathogens: *Salmonella enterica, Listeria monocytogenes*, *Staphylococcus aureus*, *Escherichia coli* O157:H7, *Shigella* spp. and *Campylobacter jejuni*. GenomeLab Gene Expression Profiler (GeXP) genetic analysis system allows high-throughput and detection of multiple pathogens in a single reaction. The analytical procedure involves primer design, PCR amplification and capillary electrophoretic separation of PCR products instead of agarose gel electrophoresis. The GeXP multiplex PCR amplification involves the use of chimeric primers, universal primers and capillary electrophoretic separation of PCR products instead of agarose gel electrophoresis. The chimeric primers contain a gene-specific sequence with a universal tag at the 5′end and they are used to produce amplicons with universal tags. Then, the universal primers which contain the same sequence as the universal tags used in the chimeric primers will drive the remaining PCR reactions. The forward universal primer is covalently labeled with a fluorescent dye at the 5′ end and it is used for the detection during capillary electrophoresis (Zhou et al., 2013). This method was found to be more sensitive and suitable for high-throughput analysis. The detection limit of GeXP-PCR for *Salmonella*, *Listeria monocytogenes*, *Staphylococcus aureus*, *Escherichia coli* O157:H7, *Shigella* spp. and *Campylobacter jejuni* was 420, 310, 270, 93, 85, and 66 CFU/mL respectively. Additional examples of the application of mPCR for the detection of foodborne pathogens in various food matrices are listed in Table 1.

**Table 1:** **Examples of the application of nucleic acid-based methods for the detection of various foodborne pathogens present in food samples**.

**Detection method**	**Foodborne pathogens**	**Detection limit**	**Food matrix**	**Assay time**	**References**
Multiplex PCR	*Salmonella* spp., *Salmonella* Enteritidis	10^3^ CFU/mL	Artificially and naturally contaminated chicken carcasses, minas cheese and fresh pork sausages	24 h	Silva et al., 2011
	STEC O26, O103, O111, O145, sorbitol fermenting O157 non-sorbitol fermenting O157	5 × 10^4^ CFU/mL in minced beef and sprouted seeds. 5 × 10^3^ CFU/mL in raw-milk cheese	Artificially contaminated minced beef, sprouted seed (soy, alfafa and leek) and raw-milk cheese	24 h	Verstraete et al., 2012
	*Escherichia coli* O157:H7, *Listeria monocytogenes Staphylococcus aureus*, *Yersinia enterocolitica*, *Salmonella*	10^3^ CFU/mL	Artificially contaminated pork	Not stated	Guan et al., 2013
Real-time PCR	*Salmonella enterica*	41.2 fg/PCR for *Salmonella* Typhimurium genomic DNA. 18.6 fg/PCR for *Salmonella* Enteritidis genomic DNA	Artificially contaminated chicken, liquid egg and peanut butter	10 h	Chen et al., 2010
	*Listeria monocytogenes*, *Escherichia coli* O157, *Salmonella* spp	<18 CFU/10 g	Artificially contaminated ground beef.	24 h	Suo et al., 2010b
			Naturally contaminated beef, pork, turkey and chicken		
	*Listeria monocytogenes*, *Escherichia coli* O157:H7, *Salmonella* spp.	2 × 10^2^ CFU/mL	Artificially contaminated ground pork	24 h	Kawasaki et al., 2010
	*Salmonella* spp., *Listeria monocytogenes*	5 CFU/25g	Artificially and naturally contaminated meat, fish, fruits, vegetables, dairy products, eggs, chocolate bar, omelet, lasagna, and various cooked dishes	<30 h	Ruiz-Rueda et al., 2011
	*Staphylococcus aureus*, *Salmonella*, *Shigella*	9.6 CFU/g for *Staphylococcus aureus*, 2.0 CFU/g for *Salmonella* and 6.8 CFU/g for *Shigella*	Fresh pork	<8 h	Ma et al., 2014
NASBA	*Escherichia coli*	40 cells/mL	Drinking water	4 h	Min and Baeumner, 2002
	*Salmonella* Enteritidis	10^1^ CFU/reaction	Artificially contaminated fresh meats, poultry, fish, ready-to-eat salads and bakery products	26 h	D'Souza and Jaykus, 2003
	*Listeria monocytogenes*	400 CFU/mL	Artificially contaminated cooked ham and smoked salmon slices	72 h	Nadal et al., 2007
	*Bacillus amyloliquefaciens, Bacillus cereus* and *Bacillus circulans*	–	Artificially contaminated milk	Not stated	Gore et al., 2003
	*Salmonella* Enteritidis and *Salmonella* Typhimurium	<10 CFU/mL	–	<90 min	Mollasalehi and Yazdanparast, 2013
LAMP	*Vibrio vulnificus*	5.4 CFU/reaction for a virulent *V. vulnificus* strain in pure culture. 2.5 × 10^3^ CFU/g for a virulent *V. vulnificus* strain in spiked raw oyster, no enrichment. 1 CFU/g for a virulent *V. vulnificus* strain in spiked raw oyster, after 6 h enrichment	Artificially contaminated raw oysters	8 h	Han et al., 2011
	*Salmonella* spp. and *Shigella* spp.	5 CFU/10 mL	Artificially contaminated milk	<20 h	Shao et al., 2011
	*Vibrio parahaemolyticus*	10 CFU/reaction	Naturally contaminated seafood samples: fish, shrimp and mussel	16 h	Wang et al., 2013a
	STEC O26, O45, O103, O111, O121, O145, and O157	1–20 cells/reaction in pure culture and 10^5^–10^6^ CFU/25 g in produce	Artificially contaminated lettuce, spinach and sprouts	Not stated	Wang et al., 2014
Oligonucleotide DNA microarray	*Escherichia coli* O157:H7, *Salmonella enterica*, *Listeria monocytogenes* and *Campylobacter jejuni*	1 × 10^−4^ ng for each genomic DNA	Naturally contaminated fresh meat samples: chicken, beef, pork and turkey	Not stated	Suo et al., 2010a
	*Listeria monocytogenes*	8 logCFU/mL	Artificially contaminated milk	Not stated	Bang et al., 2013
	*Escherichia coli*, *Shigella* spp., *Salmonella* spp., *Proteus* sp., *Campylobacter jejuni*, *Listeria monocytogenes*, *Enterococcus faecalis*, *Yersinia enterocolitica*, *Vibrio parahaemolyticus*, *Vibrio fluvialis*, *β-hemolytic Streptococcus, Staphylococcus aureus*	10 CFU/mL of pure culture	Artificially and naturally contaminated pork, chicken, fish and milk	Not stated	Huang et al., 2014

### Real-time or quantitative PCR (qPCR)

Real-time PCR or quantitative PCR is different from simple PCR whereby it does not require agarose gel electrophoresis for the detection of PCR products. This method is able to monitor the PCR products formation continuously in the entire reaction by measuring the fluorescent signal produced by specific dual-labeled probes or intercalating dyes. The fluorescence intensity is proportional to the amount of PCR amplicons (Omiccioli et al., 2009; Zhao et al., 2014).

Several fluorescent systems have been developed for qPCR and the most commonly used fluorescent systems include SYBR green, TaqMan probes and molecular beacons. SYBR green is a double-stranded DNA (dsDNA)-binding fluorescent dye (Hein et al., 2001). This non-sequence-specific intercalating dye emits little fluorescence and the fluorescence signal is enhanced when bound to the minor groove of the DNA double helix (Fukushima et al., 2003; Levin, 2005; Singh et al., 2009).

TaqMan probes and molecular beacons are the common alternatives to SYBR green. TaqMan probes, also known as double-dye probes, are oligonucleotides that contain a fluorophore as the reporter dye at the 5′-end and the quenching dye at the 3′-end (Hein et al., 2001; Levin, 2005). The reporter dye and the quenching dye are close to each other and this prevent the emitted fluorescence of the reporter (Levin, 2005). TaqMan probe is complementary to a specific nucleotide sequence in one of the strands of amplicon internal to both primers and the system depends on the 5′-3′ exonuclease activity of *Taq* DNA polymerase that cleaves the probe and separates both dyes in order to generate the fluorophore signal (Patel et al., 2006).

Molecular beacons are oligonucleotide probes with hairpin/stem-and-loop configuration, in which the sequence complementary to a target sequence is present in the loop portion and the stem is produced by annealing of two complementary arm sequences. A reporter dye is attached to one end of the probe and a quencher dye is attached to the other end. Both dyes are in close proximity which maintained by the hybrid stem, hence, no fluorescence is produced (Leone et al., 1998; Chen et al., 2000; Levin, 2005; Patel et al., 2006). Molecular beacon produces fluorescence signal upon hybridization of the probe to its complementary nucleotide sequence in the amplicon. During hybridization, the probe undergoes spontaneous conformational change that separates the two dyes and this allow fluorescence to occur (Leone et al., 1998; Chen et al., 2000; Liming and Bhagwat, 2004; Levin, 2005).

The detection of *Salmonella* in fresh-cut fruits and vegetables by molecular beacon qPCR targeting the invasion associated gene (*iagA*) was first reported by Liming and Bhagwat (2004). The detection limit was approximately 4 CFU/25 g of produce after enrichment. Tyagi et al. (2009) developed a sensitive and highly reproducible SYBR green qPCR assay for the detection of pathogenic *tdh*-positive *Vibrio parahaemolyticus* in tropical shellfish. The detection limit was 10^2^ CFU/ml for shrimp homogenates spiked with pure culture. Other advances in qPCR include the development of TaqMan and SYBR Green qPCR to identify and quantitatively detect *Staphylococcus aureus* strains that harbor the enterotoxin gene cluster (*egc*) in raw milk. Approximately 1 × 10^3^ CFU/mL *Staphylococcus aureus* was detected by SYBR green qPCR whereas 1 × 10^4^ CFU/mL *Staphylococcus aureus* was detected by TaqMan qPCR in raw milk (Fusco et al., 2011).

Furthermore, multiplex qPCR assay is also developed for the detection and quantification of multiple foodborne pathogens. Fratamico et al. (2011) performed a study to detect Shiga toxin-producing *Escherichia coli* serogroups O26, O45, O103, O111, O121, and O145 in ground beef using multiplex qPCR with primers which target Shiga toxins (*stx_1_* and *stx_2_*), intimin (*eae*) and *wzx* genes. Taqman probe with different combinations of fluorophores were used to detect the product of multiplex qPCR with detection limit around 50 CFU per PCR. Additionally, a simultaneous detection of *Vibrio parahaemolyticus, Vibrio cholerae*, and *Vibrio vulnificus* by multiplex qPCR using primers that target *vmrA, zot* and *vuuA* genes respectively was developed by Kim et al. (2012). The multiplex qPCR products was detected and quantitated by using SYBR green. Hu et al. (2014) conducted a study to detect eight foodborne bacterial pathogens which include *Salmonella enterica* subsp. *enterica*, *Listeria monocytogenes*, *Escherichia coli* O157, *Vibrio parahaemolyticus*, *Vibrio vulnificus*, *Campylobacter jejuni*, *Enterobacter sakazakii* and *Shigella* spp. by using molecular beacon based multiplex qPCR targeting *ssaR*, *hlyA*, *rfbE*, *toxR*, *vvh*, *gyrA*, 16*SrRNA* and *ipaH* genes respectively. The detection limits of the assay ranged from 1.3 × 10^3^–1.6 × 10^4^ CFU/g stool.

Among the discussed fluorescent systems for qPCR, SYBR green is simple and less costly as compared to TaqMan probes or molecular beacons (Fukushima et al., 2003; Levin, 2005). Besides, the advantage of using SYBR green is that DNA melting curve can be generated after PCR along with the calculation of the Tm value of the amplified products. This is required in order to differentiate the target products from the primer dimer formation. The primer dimer products have lower Tm values as compared to the amplicons (Levin, 2005). SYBR green lacks specificity and it binds to all double-stranded DNA, thus, it can be used to detect any PCR product (Levin, 2005; Madani et al., 2005). However, SYBR green dye will bind to other non-specific reaction products which include primer dimers (Madani et al., 2005). As for TaqMan probes and molecular beacons, they are sequence specific probes and they only bind to their target sequence, hence, primer dimers will not be detected (Levin, 2005). There are some studies have shown that TaqMan-based qPCR is more sensitive compared to SYBR green or molecular beacons-based qPCR (Hein et al., 2001; Klerks et al., 2004). Nevertheless, the sensitivity of PCR-based method is mainly affected by primer specificity, primer sequence and annealing temperature, rather than the choice of detection probe (Klerks et al., 2004). Overall, qPCR is more sensitive than conventional PCR and it minimizes the risk of cross-contamination (Omiccioli et al., 2009). More examples of the detection of foodborne bacterial pathogens by qPCR are presented in Table 1.

Conventional PCR and multiplex PCR that require agarose gel analysis for the detection of PCR products are laborious and time-consuming, thus, not suitable for high-throughput analysis and difficult to automate (Patel et al., 2006; Zhou et al., 2013). As for qPCR, post-PCR processing is not required. This allows low risk of cross-contamination, high-throughput analysis and automation (Fricker et al., 2007). The advantages of qPCR have led to the development of various commercial qPCR kits for the detection of foodborne pathogens such as *Salmonella*, *Listeria monocytogenes*, *Escherichia coli* O157:H7 and *Campylobacter* (Maurer, 2011).

The examples of commercial qPCR kits for the detection of *Salmonella* include *Salmonella* BAX™ PCR (DuPont Qualicon), AnDiaTec® *Salmonella* real time PCR Kit (AnDia Tec), Probelia™ *Salmonella* sp. (Sanofi Diagnostics Pasteur), and TaqMan™ *Salmonella* detection kit (PE Applied Biosystems) (Maciorowski, 2005; Maurer, 2011; Park et al., 2014). Kimura et al. (1999) evaluated the TaqMan™ *Salmonella* detection kit for the detection of *Salmonella* in shrimp and meat. The detection limit was 120 CFU/mL in pure culture. Wan et al. (2000) evaluated the Probelia™ *Salmonella* sp. PCR amplification and detection kits for the detection of *Salmonella agona* in artificially contaminated milk powder and ricotta cheese. The detection limit was approximately 8–79 CFU/mL. Besides, Margot et al. (2013) evaluated seven commercial qPCR kits for the detection of *Salmonella* which include BAX® system Q7 real-time PCR (DuPont Qualicon) and other commercial qPCR kit such as ADIAFOOD® *Salmonella* (AES chemunex), BIOTECON foodproof *Salmonella* Detection Kit (Biotecon Diagnostics), BioControl Assurance GDS® TM *Salmonella* (BioControl), Genedisc® Shiga Toxic *E. colii and Salmonella* spp. (Pall GeneDisc® Technologies), BioRad iQ-Check *Salmonella* 2 (Biorad) and MicroSeq® *Salmonella* spp. Detection Kit (Applied Biosystems). A total of 49 *Salmonella* strains were included in this study and correctly identified by all the evaluated systems.

The commercial qPCR kits available for the detection of *Listeria monocytogenes* are BAX® *Listeria monocytogenes* Detection System (DuPont Qualicon), TaqMan® *Listeria monocytogenes* Detection Kit (Applied Biosystems), ADIAFOOD rapid pathogen detection system for *Listeria monocytogenes* (AES Chemunex), Probelia® *Listeria monocytogenes* PCR system (Biorad), LightCycler® *Listeria monocytogenes* Detection Kit (Roche/Biotecon) and GeneVision® Rapid Pathogen Detection System for *Listeria monocytogenes* (Janzten et al., 2006; Maurer, 2011; Traunšek et al., 2011). The BAX® *Listeria monocytogenes* Detection System is adopted by the USDA (2012) as a screening test for *L. monocytogenes*. Besides, Wan et al. (2003) conducted a study to compare the Probelia® *Listeria monocytogenes* PCR system and the International Organization for Standardization (ISO) method 11290–1 for the detection of *L. monocytogenes* in salmon samples. The results indicated that Probelia® *Listeria monocytogenes* PCR system is as good as the ISO method and the detection limit was approximately 20 CFU/mL broth culture for pure culture of *L. monocytogenes.*

There are many more commercial qPCR kits that are available for the detection of other foodborne bacterial pathogens such as *Escherichia coli* and *Campylobacter*. For instance, *Escherichia coli* O157 BAX® PCR (DuPont Qualicon), ADIAFOOD rapid pathogen detection system for *E. coli* O157 and *E. coli* O157:H7 (AES Chemunex) and ADIAFOOD *Campylobacter* JCL qPCR commercial kit (AES Chemunex) for the detection of *C. jejuni*, *C. coli* and *C. lari* (Maurer, 2011; Vencia et al., 2014).

### Nucleic acid sequence-based amplification (NASBA)

NASBA is developed by Compton (1991) in the early 90s and it operates by amplifying nucleic acids under isothermal conditions, unlike PCR which requires thermocycling system. NASBA is normally used for the amplification of RNA whereby the single-stranded RNA template is converted into complementary DNA (cDNA) by the reverse transcriptase during the reaction. NASBA reaction occurs at around 41°C, involving two specific primers and three enzymes: avian myeloblastosis virus (AMV) reverse transcriptase, T7 RNA polymerase and RNase H. The NASBA amplicons can be detected by agarose gel electrophoresis (Leone et al., 1998; Zhao et al., 2014).

The post-NASBA product detection methods such as agarose gel electrophoresis or enzyme-linked gel assay is considered labor-intensive and not cost-effective. This leads to the development of a novel real-time NASBA which uses fluorescently labeled probes which are molecular beacons to detect the single-stranded RNA amplicons, thus, producing a homogenous NASBA assay (Leone et al., 1998). Real-time NASBA has been used for the detection of various foodborne pathogens such as *Salmonella enterica*, *Vibrio cholerae*, *Staphylococcus aureus, Campylobacter jejuni*, and *Campylobacter coli* (Simpkins et al., 2000; Churruca et al., 2007; Fykse et al., 2007; O'Grady et al., 2009). In addition, real-time NASBA is able to detect viable microorganisms that present in food samples through mRNA amplification and the detection of RNA targets will indicate the presence of viable cells (Simpkins et al., 2000). Real-time NASBA has been used to distinguish viable from non-viable bacterial cells and RNase treatment is usually required to degrade target mRNA from dead cells before nucleic acid extraction or treating the samples with RNase-free DNase is required prior to performing the NASBA assay (Blais et al., 1997; Nadal et al., 2007; Dwivedi and Jaykus, 2011).

NASBA offers high-throughput analysis and it has been commercialized as kits. There are several commercial NASBA kits manufactured by Life Sciences, KIT Biomedical Research, Gen-Probe and bioMérieux (Gracias and McKillip, 2007). However, the commercial NASBA kit that is used for the detection of foodborne bacterial pathogens is mainly the Nuclisens EasyQ® Basic Kit (bioMérieux). Nuclisens EasyQ® Basic Kit can be used for the detection and identification of *Listeria monocytogenes*, *Salmonella enterica* and *Vibrio cholerae* (D'Souza and Jaykus, 2003; Fykse et al., 2007; Nadal et al., 2007).

### Loop-mediated isothermal amplification (LAMP)

LAMP is a novel nucleic acid amplification method developed by Notomi et al. (2000) which provides a rapid, sensitivity and specific detection of foodborne pathogens. LAMP is based on auto-cycling strand displacement DNA synthesis carried out by *Bst* DNA polymerase large fragment under isothermal conditions between 59°C and 65°C for 60 min. In LAMP, four primers comprising two inner primers and two outer primers are used to target six specific regions of target DNA. Cauliflower-like DNA structures bearing multiple loops as well as stem-loop DNAs of different sizes are the final products of LAMP. Large amount of amplicons can be produced by LAMP within 60 min which is usually 10^3^-fold or higher as compared to simple PCR. The LAMP amplicons can be detected by agarose gel electrophoresis or SYBR Green I dye (Wang et al., 2008; Zhao et al., 2014).

In the field of foodborne pathogen detection, LAMP was used for the first time to detect *stxA_2_* gene in *Escherichia coli* O157:H7 (Maruyama et al., 2003). Since then, LAMP has been used for the detection of various foodborne pathogens due to its rapidity and sensitivity. LAMP is proven to be more specific and sensitive as compared to PCR assays for the detection of foodborne pathogens (Hara-Kudo et al., 2005; Wang et al., 2008; Yamazaki et al., 2008). This is because LAMP uses four primers targeting six specific regions and it provides rapid amplification, greater yield of amplification products and lower detection limits than PCR assays (Hara-Kudo et al., 2005; Xu et al., 2012). Till date, commercial LAMP kits are available for the detection of *Listeria, Salmonella, Campylobacter, Legionella*, and verotoxin-producing *Escherichia coli* (Mori and Notomi, 2009). For example, the Loompamp detection kit (Eiken Chemical) is commercially available for the detection of foodborne pathogens such as *Salmonella enterica* (Ohtsuka et al., 2005), *Shigella* (Song et al., 2005), enteroinvasive *Escherichia coli* (Song et al., 2005), verotoxigenic *Escherichia coli* O157 and O26 (Hara-Kudo et al., 2008) and *Campylobacter* (Yamazaki et al., 2009).

Besides, different types of LAMP assays have been developed for the detection of foodborne pathogens. For instance, multiplex LAMP, reverse-transcription LAMP, real-time LAMP and *in situ* LAMP (Chen et al., 2008; Han and Ge, 2010; Shao et al., 2011; Ye et al., 2011). The availability of real-time monitoring of LAMP amplification products by the presence of turbidity or fluorescence eliminates the need for staining with ethidium bromide and gel electrophoresis. Therefore, this allows high-throughput analysis along with its high sensitivity and specificity (Yang et al., 2010). More examples of the application of NASBA and LAMP in food samples are shown in Table 1.

### Oligonucleotide DNA microarray

The recent progress in multi-gene detection technology includes the microarray technology (Call et al., 2001). Microarrays were originally used for the study of gene expression, but oligonucleotide DNA microarray has been widely used in the field of foodborne pathogen detection. Microarrays are made up of glass slides or chips coated with up to hundreds of specific oligonucleotide probes and these probes are chemically synthesized short sequences range from 25 to 80 bp (Severgnini et al., 2011). Each oligonucleotide probe is able to target a specific part of a gene sequence. In this method, the sample nucleic acid fragments (DNA, mRNA or cDNA) are labeled with fluorescent dye, and then they are denaturated to generate single-stranded fragments. These fragments will hybridize to the array through binding to their corresponding oligonucleotide probes. The results are obtained through the visualization of the fluorescence signal produced from the probe-sample complex. The fluorescence intensity is proportional to the concentration of each labeled nucleic acid fragment (Lauri and Mariani, 2009).

Li et al. (2006) had presented the first report of detection of pathogenic *Shigella* and *Escherichia coli* serotypes by oligonucleotide DNA microarray. The detection of a specific serotype can be crucial especially for *Escherichia coli*, as this pathogen has different serotypes with different level of pathogenicity that ranges from harmless strain *Escherichia coli* K-12 to deadly strain *Escherichia coli* O157:H7 (Lauri and Mariani, 2009). Moreover, Wang et al. (2007) developed a microarray assay which allows the detection and identification of 22 foodborne pathogens. Some examples of these pathogens are *Staphylococcus aureus*, *Listeria monocytogenes*, *Vibrio parahaemolyticus*, *Vibrio cholerae*, *Campylobacter jejuni*, *Clostridium perfringens*, *Shigella* spp. *Salmonella* spp., and *Bacillus cereus*. Other studies that involved the application of oligonucleotide DNA microarray for the detection of foodborne pathogens are presented in Table 1.

DNA microarrays are commercially available but most of them are designed for gene expression analysis studies (Rasooly and Herold, 2008). The commercial *in situ*-synthesized arrays are high-density microarrays where short oligonucleotide probes range from 20 to 25 bp are synthesized directly on the surface of the microarray. In addition, multiple probes per target are included for higher sensitivity, specificity and accuracy. These high density microarrays require special manufacturing and they are relatively high in cost (Rasooly and Herold, 2008; Severgnini et al., 2011). There are various commercially available DNA microarrays manufactured by Affymetrix, Roche NimbleGen and Agilent Technologies (Severgnini et al., 2011).

Most of the commercial microarrays are not desirable for specialized application such as food microbial analysis or diagnostic laboratory because low to medium density array will serve as the ideal microarray platform that can provide reliable results without involving the use of complicated equipments and data management (Rasooly and Herold, 2008; Severgnini et al., 2011). In this case, custom microarrays are available from the Department of Bioresources at Seibersdorf and other organizations. Custom microarrays are sensitive, specific and less expensive than commercial microarrays (Mothershed and Whitney, 2006; Severgnini et al., 2011). Nevertheless, low-density microarray is commercially available. For instance, StaphyChips® developed by Affymetrix and in collaboration with Advanced Array Technology (ATT, Eppendorf Array Technologies). StaphyChips® is able to detect a total of 15 *Staphylococcus* species, including methicillin-resistant *Staphylococcus aureus* (MRSA) (Mothershed and Whitney, 2006).

In general, DNA oligonucleotide microarray allows simultaneous identification of multiple foodborne bacterial pathogens. Therefore, it is capable of high-throughput analysis and it also has the potential to be automated (Al-Khaldi, 2002; De Boer and López, 2012; Zhou et al., 2013).

## Biosensor-based methods

Biosensor is an analytical device that consists of two main elements: a bioreceptor and a transducer. The bioreceptor responsible for recognizing the target analyte can either be a:

1. Biological material: enzymes, antibodies, nucleic acids and cell receptors, or
2. Biologically derived material: aptamers and recombinant antibodies, or
3. Biomimic: imprinted polymers and synthetic catalysts.

The transducer that converts the biological interactions into a measurable electrical signal can be optical, electrochemical, mass-based, thermometric, micromechanical or magnetic (Velusamy et al., 2010; Zhao et al., 2014).

Biosensors are easy to operate and they do not require sample pre-enrichment, unlike nucleic-acid based methods and immunological methods which require sample pre-enrichment for concentrating the pathogens before detection (Singh et al., 2013). The recent biosensors that commonly used for the detection of foodborne pathogens are optical, electrochemical and mass-based biosensors (Zhang, 2013; Zhao et al., 2014). The examples of the application of different types of biosensors in foodborne pathogen detection are given in Table 2.

**Table 2:** **Examples of the application of biosensor-based methods for the detection of various foodborne pathogens present in food samples**.

**Detection method**	**Foodborne pathogens/toxin**	**Detection limit**	**Food matrix**	**Assay time**	**References**
Optical Biosensors	*Salmonella choleraesuis* serotype typhimurium, *Listeria monocytogenes*, *Campylobacter jejuni* and *Escherichia coli* O157:H7	4.4 × 10^4^ CFU/mL for *Salmonella choleraesuis* serotype typhimurium, 3.5 x 10^3^ CFU/mL for *Listeria monocytogenes*, 1.1 x 10^5^ CFU/mL for *Campylobacter jejuni* and 1.4 x 10^4^ CFU/mL for *Escherichia coli* O157:H7 in PBS	Artificially contaminated apple juice	Not stated	Taylor et al., 2006
	*Campylobacter jejuni*	10^3^ CFU/mL	Artificially contaminated broiler meat	45 min	Wei et al., 2007
	*Escherichia coli* O157:H7	3 × 10^3^ CFU/mL	Artificially contaminated cucumber and ground beef	Not stated	Wang et al., 2013b
Electrochemical Biosensors	*Escherichia coli*, *Listeria monocytogenes* and *Campylobacter jejuni*	50 cells/mL for *Escherichia coli*, 10 cells/mL for *Listeria monocytogenes* and 50 cells/mL for *Campylobacter jejuni*	Artificially contaminated milk and chicken extract	30 min	Chemburu et al., 2005
	*Escherichia coli* O157:H7	1.6 × 10^1^–7.23 × 10^7^ cells/mL without enrichment and 8.0 × 10^0^–8.0 × 10^1^ cells/mL with enrichement	Artificially contaminated ground beef	15 min without enrichment; 6 h after enrichment	Varshney et al., 2005
	*Listeria monocytogenes*	10^3^ CFU/mL	Lettuce, milk and ground beef	3 h	Kanayeva et al., 2012
Mass-based Biosensors	*Salmonella* Typhimurium	10^5^–10^6^ cells/mL	Artificially contaminated chicken meat	Not stated	Su and Li, 2005
	*Escherichia coli* O157:H7	23 CFU/mL in PBS and 53 CFU/mL in milk	Artificially contaminated milk	4 h	Shen et al., 2011

### Optical biosensors

The most commonly used optical biosensor for the detection of foodborne pathogen is surface plasmon resonance (SPR) biosensor due to their sensitivity. SPR employs reflectance spectroscopy for the pathogen detection (Velusamy et al., 2010). In SPR, bioreceptors are immobilized on the surface of a thin metal. The electromagnetic radiation of a certain wavelength interacts with the electron cloud of the thin metal and produces a strong resonance. When the pathogen binds to the metal surface, this interaction alters its refractive index which results in the change of wavelength required for electron resonance (Zhang, 2013; Zhao et al., 2014).

Commercial optical biosensors using SPR techniques such as SPREETA biosensor and BIACORE 3000 biosensor are currently available for the detection of foodborne pathogens. Waswa et al. (2007) used SPREETA biosensor for the detection of *E. coli* O157:H7 in milk, apple juice and ground beef. The detection limit was around 10^2^–10^3^ CFU/mL. Furthermore, *Salmonella* Enteritidis and *Salmonella* Typhimurium were successfully detected by SPREETA biosensor (Son et al., 2007; Lan et al., 2008). Besides, *Listeria monocytogenes* was successfully detected by BIACORE 3000 biosensor with detection limit of 1 × 10^5^ cells/mL (Leonard et al., 2004). *Salmonella* group B, D, and E, *Escherichia coli* O157:H7 and *Salmonella* Enteritidis were also successfully detected by BIACORE biosensor (Bokken et al., 2003; Waswa et al., 2006; Wang et al., 2011).

The commercially available biosensors for the detection of foodborne pathogens are mostly optical-based biosensors. The commercial biosensors offer varying degrees of automation (Leonard et al., 2003). The commercialization of biosensors is slower than other rapid methods due to several factors such as cost consideration, quality assurance, stability issues, sensitivity issues and instrumentation design. There are difficulties in the methods of producing inexpensive and reliable sensors, the storage of biosensors, the stabilization of biosensors, methods of sensor calibration and total integration of the sensor system (Velasco-Garcia and Mottram, 2003).

### Electrochemical biosensors

Electrochemical biosensors are further classified into several types such as amperometric, impedimetric, potentiometric, and conductometric according to the measurement of changes in current, impedance, voltage and conductance respectively, which caused by antigen-bioreceptor interactions (Zhang, 2013).

Many researchers had reported the successful detection of foodborne pathogens by electrochemical biosensors. For example, Pal et al. (2008) successfully detected *Bacillus cereus* present in alfalfa sprouts, strawberries, lettuce, tomatoes, fried rice and cooked corn by a direct-charge transfer conductometric biosensor. The detection limit of this method was around 35–88 CFU/mL. Amperometric magnetoimmunosensor was used for the detection of *Staphylococcus aureus*, the detection limit of this assay was 1 CFU/mL and the analysis time was 2 h (de Ávila et al., 2012). Munoz-Berbel et al. (2008) had described the use of impedimetric spectroscopy for the detection and quantification of *Escherichia coli*, the detection limit was found to be 10^1^–10^7^ CFU/mL. Ercole et al. (2003) had reported the successful detection of *Escherichia coli* in vegetable food by using antibody-based potentiometric biosensor with detection limit of 10 cells/mL.

### Mass-based biosensors

Mass-based or mass-sensitive biosensors operate based on the detection of small changes in mass. Mass-based biosensors involve the use of piezoelectric crystal which will vibrate at a certain frequency when induced by an electrical signal of a certain frequency. The bioreceptors (e.g., antibodies) for the detection of pathogens (e.g., antigens) are immobilized on this crystal. Once the target antigens bind to the antibodies immobilized on the crystal, this will cause a measurable change in the vibrational frequency of the crystal which correlates with the added mass on the crystal surface. There are two major types of mass-based biosensors which are the bulk acoustic wave resonators (BAW) or quartz crystal microbalance (QCM) and surface acoustic wave resonators (SAW) (Velusamy et al., 2010; Zhang, 2013).

However, the application of mass-based biosensors in the field of foodborne pathogen detection is generally lesser than electrochemical and optical biosensors (Velusamy et al., 2010). The use of piezoelectric immunosensor for the detection of *Salmonella* Enteritidis with detection limit of 1 × 10^5^ cells/mL (Si et al., 2001) and the detection of *Escherichia coli* with detection limit of 10^6^–10^9^ CFU/mL (Pohanka et al., 2007). Detection of toxigenic *Escherichia coli* O157:H7 by using a SAW biosensor was reported by Berkenpas et al. (2006). The QCM immunosensor was employed by Vaughan et al. (2001) for the detection of *Listeria monocytogenes* and the detection limit was 1 × 10^7^ cells/mL. Moreover, Liu et al. (2007) employed QCM immunosensor for the detection of *Escherichia coli* O157:H7 and the detection limit was 10^2^ CFU/mL with detection time of less than 1.5 h.

## Immunological-based methods

The detection of foodborne pathogens by immunological-based methods is based on antibody-antigen interactions, whereby a particular antibody will bind to its specific antigen. The binding strength of a particular antibody to its antigen determines the sensitivity and specificity of immunological-based methods. Immunological-based methods involve the use of polyclonal and monoclonal antibodies (Zhao et al., 2014). Enzyme-linked immunosorbent assay (ELISA) and lateral flow immunoassay is among the immunological-based methods which recently used for the detection of foodborne pathogens.

### Enzyme-linked immunosorbent assay (ELISA)

ELISA is one of the most commonly used immunological methods for the detection of foodborne pathogens. Sandwich ELISA is the most effective form of ELISA whereby it involves two antibodies (Zhao et al., 2014). The primary antibody is usually immobilized onto the walls of the microtiter plate wells. The target antigen like bacterial cells or bacterial toxins from the food sample binds to the immobilized primary antibody and the remaining unbound antigens are removed. After that, an enzyme-conjugated secondary antibody is added and it will bind to the antigen and the remaining unbound antibodies are removed. The complex consisting antigen sandwiched between two antibodies is formed and it can be detected by adding a colorless substrate which will be converted into a colored form in the presence of the enzyme (Zhang, 2013). There are different types of enzymes can be used in ELISA, some of the most commonly used enzymes include horseradish peroxidase (HRP), alkaline phosphatase and beta-galactosidase (Yeni et al., 2014).

Many studies have been performed using the sandwich ELISA for rapid detection of foodborne pathogens. For example, Kumar et al. (2011) performed the detection of pathogenic *Vibrio parahaemolyticus* in seafood with sandwich ELISA, using monoclonal antibodies against the TDH-related hemolysin (TRH) of pathogenic *Vibrio parahaemolyticus*. The detection limit of this assay was 10^3^ cells of pathogenic *Vibrio parahaemolyticus*. Commercial ELISA test kit such as BIOLINE *Salmonella* ELISA Test is also available for the detection of *Salmonella* in food products. The detection limit of this test kit was 1 CFU/25 g sample with minimum four of the 20 food matrixes tested (Bolton et al., 2000). ELISA is also commonly used for the detection of toxins present in foods such as *Clostridium perfringens* α, β, and ε toxin, staphylococcal enteroxins A, B, C, and E, botulinum toxins and *Escherichia coli* enterotoxins (Aschfalk and Mülller, 2002; Zhao et al., 2014).

Recently, high-throughput and automated ELISA systems such as VIDAS (BioMerieux) and Assurance EIA (BioControl) are available for the detection of foodborne pathogens (Glynn et al., 2006). VITEK immunodiagnostic assay system (VIDAS) is system that performs entire ELISA procedure automatically. It utilizes enzyme-linked fluorescent immunoassay (ELFA) which is similar to ELISA, but it is a more sensitive fluorescent immunoassay for reporting the results. Generally, this system can complete an assay in 45 min to 2 h which also depends on the test kit. The VIDAS system involves the use of reagent strip and a plastic tube known as solid phase receptacle (SPR). A liquid sample of an enriched sample is placed in the reagent strip that contains all the required reagents in a ready-to-use format. The SPR serves as the pipette and the solid phase for the assay. The instrument will perform ELFA by automatically transferring the sample to the SPR that coated with antibodies in its interior wall in order to capture the target pathogen or toxin. The SPR is then automatically transferred to a series of wells that contain enzyme-conjugated secondary antibodies and enzymes in a sequence manner. Once the assay is completed, the result will be automatically analyzed by the instrument and interpreted as positive or negative (Vaz-Velho et al., 2000; Fung, 2002).

Several studies applied VIDAS for the detection of *Salmonella* in pork sample, fruits and vegetables (Vieira-Pinto et al., 2007; Gómez-Govea et al., 2012), *Listeria monocytogenes* in fish samples, beef, pork, fruits, and vegetables (Vaz-Velho et al., 2000; Meyer et al., 2011; Gómez-Govea et al., 2012), *Escherichia coli* O157:H7 in Minas Frescal cheese, fruits, and vegetables (Gómez-Govea et al., 2012; Carvalho et al., 2014), *Campylobacter* spp. in fruits and vegetables (Gómez-Govea et al., 2012), and staphylococcal enterotoxin in raw milk cheese (Cremonesi et al., 2007).

Assurance Enzyme Immunoassay (EIA) is a commercial ELISA kit that allows automation and high-throughput testing (Fung, 2002). The Assurance EIA test kits for *Salmonella, Escherichia coli*, O157:H7, *Listeria monocytogenes*, and *Campylobacter* are currently available (Fung, 2002). Assurance EIA test kit for the detection of *Escherichia coli* O157:H7 in raw and cooked beef was used in the study conducted by Feldsine et al. (2002). Besides, Stewart et al. (2001) performed the detection of *Salmonella* in alfalfa sprouts by Assurance EIA test kit and Taha et al. (2010) performed the detection of *Salmonella* in chicken meat by Assurance EIA test kit.

### Lateral flow immunoassay

ELISA offers a sensitive and accurate detection of foodborne pathogens. However, the operation of ELISA requires specialized equipment and trained personnel (Zhao et al., 2014). Hence, other immunological detection methods which are rapid, cheap, simple and reliable are required. Lateral flow immunoassays such as dipstick and immunochromatographic strips have been developed for rapid on-site detection of foodborne pathogens. Lateral flow immunoassay device is made up of four sections which are arranged orderly on a plastic backing, with sample pad starting at the bottom, followed by conjugate pad, nitrocellulose membrane and then absorbent pad. The sample fluid will migrate along the four sections of lateral flow immunoassay via capillary action. The sample fluid encounters and mixes with the conjugate, which can be antibody or antigen labeled by a color particle, at the conjugate pad and then pass through the lines in the nitrocellulose membrane that immobilized with antibody or antigen. The color particle can bind to the antibody or antigen immobilized at test line depending on the analytes present in the sample. The color can be visualized approximately two to 10 min after the addition of sample. There are two basic formats of lateral flow immunoassays: competitive assay which used to test analytes with single epitope and sandwich assay which used to test analytes with several epitopes (Ngom et al., 2010; Zhao et al., 2014).

The detection of foodborne pathogens by lateral flow immunoassay employs labels such as monodisperse latex, colloidal gold, carbon and fluorescent tags (Zhao et al., 2014). Immunochromatographic strip developed by Jung et al. (2005) to detect *Escherichia coli* O157 in enriched samples had used colloidal gold particles as label. This study had showed that the detection limit for *Escherichia coli* O157 without enrichment was 1.8 × 10^5^ CFU/mL and after enrichment was 1.8 CFU/mL. Niu et al. (2014) employed an immunochromatographic test strip based on sandwich format with colloidal gold as label for the detection of *Staphylococcus aureus*. The detection limit for *Staphylococcus aureus* the study was 10^3^ CFU/mL. Moreover, Xu et al. (2013) developed a novel immunochromatographic strip test which based on sandwich format with fluorescent microspheres as label for the detection of *Campylobacter jejuni*. The detection limit of this test was 10^6^ CFU/mL. Lateral flow immonuassay is also used for detection of other foodborne bacterial pathogens such as *Listeria* spp. and *Salmonella* (Kim et al., 2007; Shukla et al., 2011). It can also be used to detect toxins which may cause foodborne diseases such as brevetoxins and staphylococcal enterotoxin B (Zhou et al., 2009; Rong-Hwa et al., 2010).

Commercial immunochromatographic test strips are also available for the detection of foodborne bacterial pathogens. For instance, Reveal® test kits (Neogen) for *Listeria, Salmonella* and *Escherichia coli* O157, VIP® GOLD™ (BioControl Systems) for *Listeria, Salmonella*, and *Escherichia coli* and DuPont™ Lateral Flow System (DuPont Qualicon) for *Listeria* (Fung, 2002; Shukla et al., 2011; Cho and Irudayaraj, 2013; Leem et al., 2014). Lateral flow immunoassays are simple and fast, but they are designed for individual tests rather than high-throughput screening (De Boer and López, 2012). More examples of the application of immunological-based methods for the detection of foodborne pathogens in different food matrices are presented in Table 3.

**Table 3:** **Examples of the application of immunological-based methods for the detection of various foodborne pathogens present in food samples**.

**Detection method**	**Foodborne pathogens/toxin**	**Detection limit**	**Food matrix**	**Assay time**	**References**
ELISA	*Escherichia coli* O157:H7	68 CFU/mL in PBS and 6.8 × 10^3^ CFU/mL in food samples	Artificially contaminated milk, vegetable and ground beef	3 h	Shen et al., 2014
Lateral Flow Immunoassay	*Salmonella* Typhi	10^4^–10^5^ CFU/mL	Artificially contaminated food rinses (meat, chicken and vegetables) and milk	10 h	Kumar et al., 2008
	*Salmonella* Typhimurium	30 cells/25 g	Artificially contaminated tomato samples	Not stated	Shukla et al., 2014

## Advantages and limitations of rapid methods

Rapid methods provide various advantages for the detection of foodborne pathogens, however, they also have several limitations as summarized in Table 4.

**Table 4:** **The summary of advantages and limitations of each rapid detection methods**.

**Detection method**		**Advantages**	**Limitations**	**References**
Nucleic acid-based	Simple PCR	• High sensitivity	• Affected by PCR inhibitors, Requires DNA purification	Mandal et al., 2011; Zhang, 2013; Park et al., 2014
		• High specificity	• Difficult to distinguish between viable and non-viable cells	
		• Automated		
		• Reliable results		
	Multiplex PCR	• High sensitivity	• Affected by PCR inhibitors	Mandal et al., 2011; Zhang, 2013; Park et al., 2014
		• High specificity	• Difficult to distinguish between viable and non-viable cells	
		• Detection of multiple pathogens	• Primer design is crucial	
		• Automated		
		• Reliable results		
	Real-time PCR	• High sensitivity	• High cost.	Mandal et al., 2011; Zhang, 2013; Park et al., 2014
		• High specificity	• Difficult for multiplex real-time PCR assay	
		• Rapid cycling	• Affected by PCR inhibitors.	
		• Reproducible	• Difficult to distinguish between viable and non-viable cells	
		• Does not require post-amplification products processing	• Requires trained personnel.	
		• Real-time monitoring PCR amplification products	• Cross contamination may occur	
	NASBA	• Sensitive	• Requires viable microorganisms	Lauri and Mariani, 2009; Zhao et al., 2014
		• Specific	• Difficulties in handling RNA	
		• Low cost		
		• Does not require thermal cycling system		
		• Able to detect viable microorganisms		
	LAMP	• High sensitive	• Primer design is complicated	Zhao et al., 2014
		• High specificity	• Insufficient to detect unknown or unsequenced targets	
		• Low cost		
		• Easy to operate		
		• Does not require thermal cycling system		
	Oligonucleotide DNA microarray	• High sensitivity	• High cost	Lauri and Mariani, 2009; Mandal et al., 2011; Park et al., 2014
		• High specificity	• Difficult to distinguish between viable and non-viable cells	
		• High throughput	• Requires trained personnel	
		• Enables detection of multiple pathogens	• Requires oligonucleotide probes and labeling of target genes	
		• Allows detection of specific serotype		
		• Labor-saving		
Biosensor-based	Optical biosensors	• High sensitivity	• High cost	Ivnitski et al., 1999; Mandal et al., 2011; Zhang, 2013
		• Enables real-time or near real-time detection		
		• Label-free detection system		
	Electrochemical biosensors	• Can handle large numbers of samples	• Low specificity	Ivnitski et al., 1999; Mandal et al., 2011; Zhang, 2013
		• Automated	• Not suitable for analyzing samples with low amount of microorganisms	
		• Label-free detection	• Analysis may interfered by food matrices	
			• Many washing steps	
	Mass-based biosensors	• Cost effective	• Low specificity	Ivnitski et al., 1999; Mandal et al., 2011; Zhang, 2013
		• Easy to operate	• Low sensitivity	
		• Label-free detection	• Long incubation time of bacteria	
		• Real-time detection	• Many washing and drying steps	
			• Regeneration of crystal surface may be problematic	
Immunological-based	ELISA	• Specific	• Low sensitivity	
		• Can be automated so that it is more time efficient and labor-saving	• False negative results	
		• Allows the detection of bacterial toxins	• May result in cross-reactivity with closely related antigens	
		• Can handle large numbers of samples	• Pre-enrichment is required in order to produce the cell surface antigens	Zhang, 2013; Park et al., 2014; Zhao et al., 2014
			• Requires trained personnel	
			• Requires labeling of antibodies or antigens	
	Lateral flow Immunoassay	• Low cost	• Requires labeling of antibodies or antigens	Zhao et al., 2014
		• Reliable		
		• Easy to operate		
		• Sensitive		
		• Specific		
		• Allow the detection of bacterial toxins		

## Conclusion

Conventional methods for the detection of foodborne pathogens which based on culturing the microorganisms are selective, but they can be time-consuming and laborious. Hence, various rapid detection methods have been developed in order to overcome the limitations of conventional detection methods. Rapid methods are important for the rapid detection of foodborne pathogens in food products to prevent outbreaks of foodborne diseases and the spread of foodborne pathogens. Rapid detection methods are generally more sensitive, specific, time-efficient, labor-saving, and reliable than conventional methods.

Nucleic acid-based methods such as PCR, mPCR, qPCR, and DNA microarray have high sensitivity and they are widely used for the detection of foodborne pathogens, but these methods require trained personnel and specialized instruments. Alternative nucleic acid-based methods such as NASBA and LAMP are available for the detection of foodborne pathogens and their toxins. NASBA and LAMP are relatively sensitive, specific and cost efficient. They do not require thermocycling system therefore they are useful especially in low resource settings. Furthermore, numerous biosensors-based methods have recently emerged and employed in the field of foodborne pathogen detection due to their rapidness and cost effectiveness. Biosensors-based methods are easy to operate and they do not require trained personnel, furthermore the techniques can be used for the detection of foodborne pathogens without sample pre-enrichment. However, improvement in food matrixes detection is still needed for these methods for on-site detection. Immunological-based methods such as ELISA and lateral flow immunoassay are also used for the detection of foodborne bacterial pathogens and their toxins. Immunological methods work best in the absence of interfering molecules in the samples such as non-targeted cells, DNA or proteins. Combination of several rapid methods for the detection of a particular foodborne pathogen is also possible as the use of only one detection method may not be sufficient to confirm the detected pathogen. Further studies on the effect of different combinations of rapid methods for foodborne pathogen detection are required in order to develop the most effective and accurate detection method.

### Conflict of interest statement

The authors declare that the research was conducted in the absence of any commercial or financial relationships that could be construed as a potential conflict of interest.
